# Long-Term Clinical Behavior and Complications of Intentionally Tilted Dental Implants Compared with Straight Implants Supporting Fixed Restorations: A Systematic Review and Meta-Analysis

**DOI:** 10.3390/biology10060509

**Published:** 2021-06-08

**Authors:** Jorge Cortés-Bretón Brinkmann, Ignacio García-Gil, Patricia Pedregal, Jesús Peláez, Juan Carlos Prados-Frutos, María Jesús Suárez

**Affiliations:** 1 Department of Dental Clinical Specialties, Faculty of Dentistry, Complutense University of Madrid, 28040 Madrid, Spain; jcortesb@ucm.es; 2 Department of Conservative Dentistry and Orofacial Prosthodontics, Faculty of Dentistry, Complutense University of Madrid, 28040 Madrid, Spain; iggarc05@ucm.es (I.G.-G.); patriciapedregal@ucm.es (P.P.); mjsuarez@odon.ucm.es (M.J.S.); 3 Department of Medical Specialties and Public Health, Rey Juan Carlos University, 28922 Alcorcón, Spain; prof.prados@gmail.com; 4 IDIBO GROUP (Group of High-Performance Research, Development and Innovation in Dental Biomaterials), Rey Juan Carlos University, 28922 Alcorcón, Spain

**Keywords:** systematic review, dental implants, axial, tilted, marginal bone loss

## Abstract

**Simple Summary:**

Following tooth loosening due to periodontal disease, caries, trauma, or tumoral processes, bone resorption and remodeling of the alveolar ridge makes the insertion of implants difficult. A number of bone augmentation techniques are available to treat atrophic jaws. However, when posterior bone is lacking and extensive bone augmentation surgeries are rejected by the patient, placing distally tilted posterior implants may offer a valid therapeutic option for implant-supported restorations. This treatment modality places the implants in preexisting bone, improving bone anchorage and prosthetic support. Nevertheless, some studies suggest that for tilted implants, bending moments are greater at the level of the angled abutment.

**Abstract:**

The aim of this study was to assess the long-term clinical behavior of straight implants in comparison with intentionally tilted dental implants (ITDI) supporting fixed restorations in partial or total edentulous arches, analyzing implant survival and success rates, complications, and marginal bone loss (MBL) after >5 years of function. An electronic search was conducted in five electronic databases (MEDLINE/Pubmed, Embase, Web of Science, Scopus, and Cochrane Central Register of Controlled Trials) supplemented by a manual search. The electronic and manual search identified 1853 articles, of which 8 articles were selected for analysis. Out of a total of 3987 dental implants, 2036 were axial dental implants and 1951 tilted. Similar results were found in implant survival or overall implant success rates. Moreover, no statistically significant differences were found in MBL (*p* = 0.369; MD 0.116 mm (−0.137; 0.369) 95% CI) The prosthodontic/biological complications reported in the articles were very diverse and irregularly distributed. This systematic review suggests that there is no difference between tilted compared with straight dental implants in the medium-long term (>5 years). However, further research is needed to generate long-term data and confirm the present review’s findings.

## 1. Introduction

The rehabilitation of totally or partially edentulous jaws by means of osseointegrated implants has proven to be a predictable treatment option over time [1], enjoying success rates of between 92.5% and 96.4% and a survival rate of 94.7–99.4% over at least 5 years [2,3,4]. However, rehabilitation is often limited by a scarcity of residual bone or poor bone quality, especially in the premolar-molar region. Pneumatization of the maxillary sinus and the presence of the mandibular nerve also present challenges when it comes to restoring edentulous patients. In cases of atrophic maxillae and/or mandibles, several treatment options are available to make implant placement possible, including guided bone regeneration, split crest technique, sinus lift, osteogenic distraction, or bone blocking, among others [5,6].

When posterior bone is lacking and extensive bone augmentation surgeries are rejected by the patient, implant placement often results in a longer distal cantilever that produces high stress on both implants and bone. This can compromise implant survival [7]. In such cases, short implants or implant placement in the zygoma or the tuberosity offer an alternative to advanced bone-augmentation surgeries [8,9,10].

Another option is to place a distally tilted posterior implant. This treatment modality places the implants in preexisting bone, improving bone anchorage and prosthetic support. Tilting the implants may have other advantages too, such as the possibility of placing long implants, which increases the bone-to-implant contact area as well as primary stability. It also increases the distance between anterior and posterior implants, which results in better load distribution and avoids long cantilevers [11,12,13].

Nevertheless, some studies suggest that for tilted implants, bending moments are greater at the level of the angled abutment. However, the rigidity of the prosthesis and increased prosthetic support may keep stress to the implants and the adjacent bone within acceptable levels [10,11,12,13].

To our knowledge, no systematic review to date has evaluated the long-term clinical behavior of intentionally tilted dental implants (ITDI). Therefore, the objectives of this review were to determine the clinical behavior of axial implants compared with intentionally tilted implants supporting fixed restorations in partial or total edentulous arches in terms of implant success and survival rates, complications, and peri-implant marginal bone loss after at least 5 years of function.

## 2. Materials and Methods

### 2.1. Protocol Development and PICO Question

This systematic review followed guidelines established in the PRISMA (Preferred Reporting Items for Systematic Review and Meta-Analyses) statement [14,15], using the following PICO (Population, Intervention, Comparison, Outcome) model:

- Population: Systemically healthy edentulous and partially edentulous patients.
- Intervention: Implant-supported restorations with tilted dental implants.
- Comparison: Implant-supported restorations with axial dental implants.
- Outcome: Long-term clinical behavior, focusing on implant success and survival rates, peri-implant marginal bone loss, and prosthodontic/biological complications around tilted implants in comparison with axial implants.

Therefore, the PICO question was: In edentulous and partially edentulous patients (P), what are the implant success and survival rates, prosthodontic/biological complication rates, and marginal bone loss (O) of intentionally tilted placed dental implants (I) in comparison with axial dental implants (C)?

### 2.2. Eligibility Criteria

#### 2.2.1. Inclusion Criteria

- Clinical human studies of implant-supported restorations comparing intentionally tilted placed dental implants (ITDI) with axial dental implants.
- Types of study: randomized controlled clinical trials, cohort studies, clinical trials, multi-center studies, pragmatic clinical trials, case-control studies.
- Clinical human studies reporting data on: implant survival rates, implant success rates, marginal bone loss, prosthodontic/biological complications of axial and tilted dental implants.
- Follow-up of at least five years.
- Total number of patients/study arm or cohort greater than 25.
- Articles published in English, Spanish, or German
- Human studies published during the last 10 years.

#### 2.2.2. Exclusion Criteria

- Clinical studies that did not provide >5-year follow-up.
- Clinical studies that did not compare axial and tilted dental implants within the same study.
- Studies of zygomatic implants.
- Animal studies or in vitro studies.
- Review articles or case reports.

### 2.3. Type of Intervention and Comparisons

The review selected studies of interventions for treating edentulous and partially edentulous patients by means of fixed restorations supported by tilted implants compared with axial implants.

### 2.4. Data Collection

The primary outcomes used to evaluate the long-term clinical behavior of angled vs. axial dental implants supporting fixed restorations were implant survival and success rates. The following secondary outcomes were also analyzed: marginal bone loss around dental implants and prosthodontic/biological complications.

Implant survival was understood as cases in which an implant was in function with no mobility or suppuration. Implant success also included a radiographic bone loss less than 2 mm compared with the marginal bone level at the moment of implant insertion surgery [16], no evidence of peri-implant radiolucency in radiographs, no suppuration or pain at the implant site, or ongoing pathologic processes.

### 2.5. Sources an Search Strategy

An electronic search was conducted in five electronic databases: The National Library of Medicine (MEDLINE/Pubmed); Embase; Web of Science; Scopus; and Cochrane Central Register of Controlled Trials. Two independent researchers (I.G.-G. and P.P.) carried out the search. The search strategy sought to locate studies published in English, Spanish, and German published between 1 January 2010 and 2 November 2020. An additional manual search was also conducted in the same period of time in the following journals: Clinical Implant Dentistry and Related Research, International Journal of Oral and Maxillofacial Implants, Journal of Oral Implantology, Clinical Oral Implants Research, Journal of Dental Research, European Journal of Oral Implantology, Implant Dentistry, International Journal of Oral and Maxillofacial Surgery, Journal of Prosthodontic Research, Journal of Dentistry, Clinical Oral Investigations, Journal of Prosthodontics-Implant Esthetic and Reconstructive Dentistry, Journal of Prosthetic Dentistry, and Journal of Oral Rehabilitation.

The digital search terms applied were as follows: (Dental implant AND (straight OR axial)) AND/OR (Dental implant AND (tilted OR tipped OR angulated OR tilting OR tipping)). The search was conducted in advance mode with no filters.

### 2.6. Study Selection, Screening Methods, and Clinical Data Extraction

Two reviewers (I.G.-G. and P.P.) working independently screened the titles and abstracts of the articles located in the electronic and manual searches after discarding any duplicates. When data were incomplete or missing from an article, the authors were contacted. If any doubt arose, data were excluded until further clarification was available. The same reviewers read the full manuscripts of all articles that fulfilled the inclusion criteria, as well as any manuscripts without sufficient data in the title and abstract on which to base a decision. Any disagreement was resolved by discussion with a third reviewer (J.C.-B.B.).

Inter-reviewer reliability (percentage of agreement and kappa correlation coefficient) in the selection process and after full text analysis was calculated.

If studies with the same patient cohort were recorded in several studies, only the study with the longest follow-up period was included. In cases of multiple cohorts in the same study, clinical data from each group were recorded separately.

If studies incorporated data of patients with different follow-up times, only data from the last time frame (>5 years) were collected.

### 2.7. Risk of Bias in Individual Studies

The version 2 of the Cochrane risk-of-bias tool (RoB2) for randomized trials was used to assess the risk of bias in RCTs [17]. RoB2 is structured into a fixed set of domains of bias, focusing on different aspects of trial design, conducting, and reporting. Within each domain, a series of questions (‘signaling questions’) aim to elicit information about features of the trial that are relevant to risk of bias.

The Newcastle–Ottawa scale (NOS) was used to assess the quality of cohort studies, observational studies, and non-randomized trials [18]. The NOS calculates study quality on the basis of three major components: selection, comparability, and outcome. Each individual study received a maximum of 9 points.

### 2.8. Statistical Analysis

Marginal bone loss around axial and tilted dental implants were calculated with a confidence interval (CI) of 95% using fixed or random-effects models depending on the heterogeneity of the trials (represented by means of forest plots). Cochran’s Q test and I^2^ test were used to determine statistical heterogeneity. A random-effects model was applied when the level of heterogeneity was not regarded as within acceptable limits (I^2^ value 50–100%; Q test (*p* < 0.05)) [19,20]. Data were entered in a Microsoft TM Excel spreadsheet, and analysis was carried out using Stata SE Version 15 statistical software (Stata Corp LLC, College Station, TX, USA).

## 3. Results

### 3.1. Study Selection

**Figure 1:**
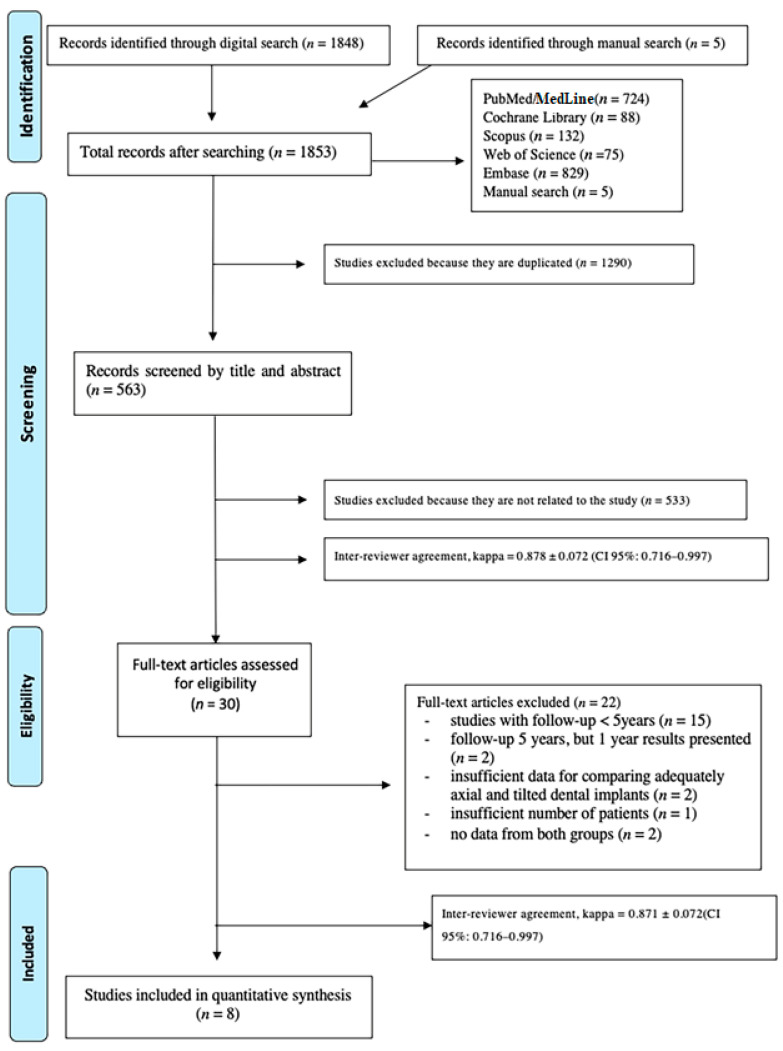
Flow chart of the inclusion and exclusion of studies in this review.

The initial electronic database search yielded 1848 articles and the manual search identified five more. Of these studies, 1290 were duplicates and were removed. After screening all titles and abstracts, the full texts of 30 articles were read. Finally, a total of 8 studies fulfilled the inclusion criteria and were selected for data extraction and analysis. A flow diagram (Figure 1) illustrates the entire search and selection process.

### 3.2. Study Characteristics

Information about the studies selected including study design, study objectives, sample size, assessment methodology, follow-up period, and other data is shown in Table 1, Table 2 and Table 3. Four articles were prospective cohort studies (21–24) and four were retrospective cohort studies (25–28). All the studies had follow-up periods of at least 5 years and the maximum follow-up period was 17 years.

### 3.3. Study Characteristics

Only cohort studies were included in the present systematic review. Therefore, quality assessment was made by means of the Newcastle–Ottawa scale, obtaining scores of 3 to 6 points (Table 4). The assessed studies obtained scores of 6 points in one study (medium risk of bias) [23], six studies obtained 5 points (medium risk of bias) [21,24,25,26,27,28], and one study obtained 3 points (high risk of bias) [22].

### 3.4. Synthesis of Results

#### 3.4.1. Inter-Reviewer Agreement

The inter-reviewer Kappa statistic between the two independent reviewers (I.G.-G. and P.P.) for title and abstract selection was 0.878 ± 0.072 (CI 95%) and 0.871 ± 0.072 (CI 95%) for the full text assessment inclusion. The intervention of a third reviewer for consensus purposes was not needed.

#### 3.4.2. Patient Characteristics

A total of 1113 patients with a mean age of 61.71 years were included in the studies. Follow-up periods ranged from 5 to 17 years. The proportion of men to women was 1:44 (478 men and 686 women) and of smokers to non-smokers 1:3.08 (259 smokers and 799 non-smokers). The total number of dental implants evaluated after >5 years was 3987, of which 2036 implants were axial and 1951 tilted.

#### 3.4.3. Marginal Bone Loss

**Figure 2:**
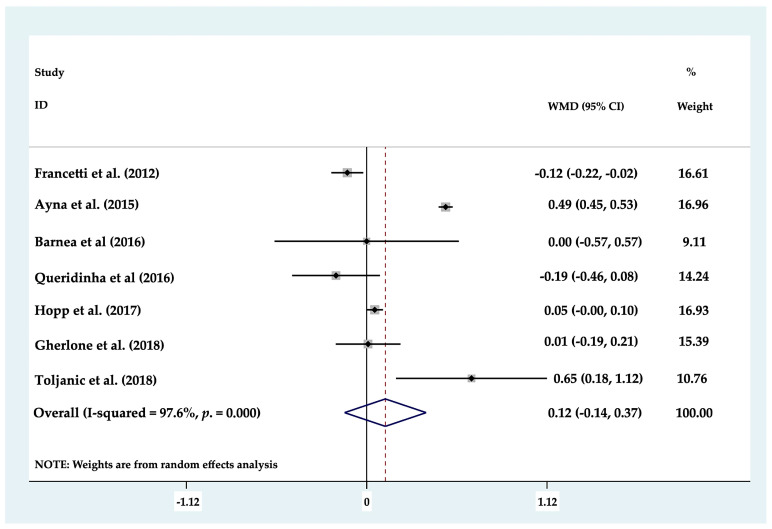
Forest plot of marginal bone loss. No statistically significant differences (*p* ≥ 0.05) were found.

**Figure 3:**
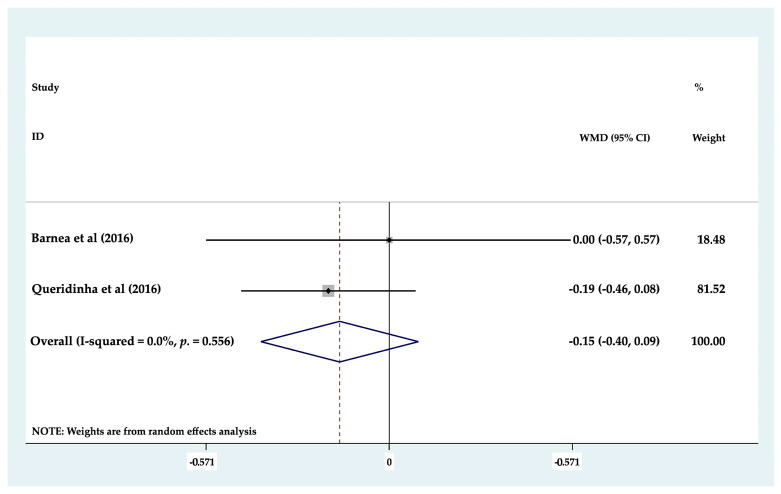
Forest plot of marginal bone loss in partial arch restorations. No statistically significant differences (*p* ≥ 0.05) were found.

**Figure 4:**
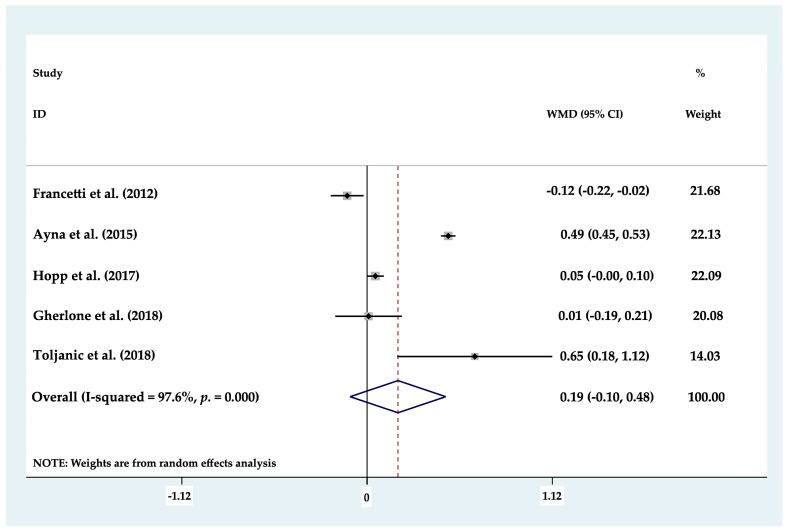
Forest plot of marginal bone loss in full arch restorations. No statistically significant differences (*p* ≥ 0.05) were found.

Of the eight clinical studies analyzed, only seven studies could be included in statistical analysis of MBL, as these works evaluated MBL around axial (mean 1.17 mm) and tilted (mean 1.2834 mm) dental implants separately. Forest plots were produced to compare marginal bone loss between axial and tilted implants supporting partial or full rehabilitations; the results found no statistically significant differences (*p* ≥ 0.05) between the two groups (*p* = 0.369; MD 0.116 mm (−0.137–0.369) 95% CI) (Figure 2). To determine whether there was any difference between the different types of prosthetic restoration (full or partial), each group was analyzed but again, no statistically significant difference (*p* ≥ 0.05) was identified between tilted and axial implants supporting partial restorations, (*p* = 0.217; MD −0.155 mm (−0.401–0.091) 95% CI) (Figure 3) or full arch restorations (*p* = 0.205; MD 0.186 mm (−0.102–0.475) 95% CI) (Figure 4).

#### 3.4.4. Implant Survival Rates

A total of 3987 dental implants were placed in the studies analyzed, obtaining 96.66% survival and 3.34% failure rates for axial implants and 96.93% survival and 3.072% failure for tilted implants. In this case, meta-analyses were not performed since only two studies could be evaluated due the heterogeneity of the studies.

Different implant survival criteria were applied in the articles reviewed. Francetti et al. [21] considered implant survival as no evidence of peri-implant radiolucency in radiographs, no suppuration or pain at the implant site, and no pathologic processes. This was similar to Agnini et al. [22], although these authors also applied the criterion that implants must be stable and in function. For Guerlone et al. [23], implant survival was defined as the absence of implant mobility, swelling, or pain at the surgical site at the time of examination. Toljanic et al. [25] defined implant survival as the absence of untreatable progressive marginal bone loss, pain, and/or infection.

#### 3.4.5. Implant Success Rates

Only three clinical studies evaluated implant success rates, obtaining a mean success rate of 95.1% for axial and 96.36% for tilted implants [24,26,27]. It should be noted that the criteria applied to evaluate success rates were not exactly the same in all the studies.

Ayna et al. [24] used the current success criteria established by the Pisa consensus [16]. Bone loss was more pronounced around distal, tilted implants, but losses fell within the 2 mm threshold considered to constitute ‘success,’ by a minimum margin of at least 0.5 mm in 100% of the implants.

Barnea et al. [26] obtained comparable success rates for tilted and axial implants applying Albrektsson and Zarb criteria [29]. The success rate was 89.6% (26 out of 29 implants) for axial and 93.1% (27 out of 29) for tilted implants.

Hopp et al. [27] based implant success on the Malo Clinic success criteria: implant in function supporting a prosthesis, without individual mobility, no signs of infection, no radiolucent areas, good rehabilitation aesthetics, and satisfactory prosthetic hygiene and comfort. The article reported success rates of 96.1% and 95.7% for tilted and axial implants, respectively. Implant failure was found to be statistically independent of implant orientation (tilted or axial). Queridinha et al. [28] and Guerlone et al. [23] applied specific implant success criteria but only provided the survival rates in their results (without detailing the criteria applied to determine survival).

#### 3.4.6. Dental Implant Angulation and Abutments

Of the 10 clinical studies reviewed, seven used abutments with disparate angulations of 0°, 15°, 17°, 25°, or 30°, while the remaining three studies did not specify abutment angulation.

Analyzing implant axis angulation in comparison with axial implants, seven studies reported multiple angles of divergence (20°, 30°, 40°, 45°, and 50°), while one study did not provide these data [25]. Only three studies reported the methods used to measure these angulations (with guide, splint, or intraoral x-ray) [21,25,26].

#### 3.4.7. Properties of Dental Implants and Loading

Two of the most commonly evaluated implant characteristics were length (ranging from 7 to 18 mm) and width (3.3–5 mm). Regarding the type of loading performed, most studies used the immediate loading protocols (*n* = 7), while others delayed loading (*n* = 1) [26].

#### 3.4.8. Restoration Type and Location

The studies reviewed described a wide variety of prosthetic restorations. Four studies placed fixed metal-ceramic prostheses, two studies fixed metal-acrylic prostheses, two placed hybrid titanium frameworks with composite, another study delivered fixed metal-ceramic and acrylic prostheses, and the last loaded the implants with CAD-CAM Proceram prostheses.

Regarding the types of restoration performed, most of the studies (*n* = 6) performed full arch restorations, while two rehabilitated patients were treated with fixed partial dentures (*n* = 2) [26,28]. The location of the dental implants according to arch were classified as maxillary, mandibular, or both. Five studies restored edentulous maxillas (*n* = 5), of which three described full arch restorations, while the other two articles carried out partial restorations in the posterior regions. Two studies performed mandibular full arch restorations (*n* = 2), while the remaining three articles carried out full arch restorations in either arch.

#### 3.4.9. Complications

The studies reported different types of both prosthetic and peri-implant complications. Regarding mechanical complications, three studies (*n* = 3) evaluated prosthetic survival rates (Barnea et al. 100% [26]; Hopp et al. 99.8% [27]; Gherlone et al. 100% [23]). Screw loosening (*n* = 4) was the most widely reported complication with the following results: Ayna et al. 0.92% of dental implants [24]; Barnea et al. 3.4% [26]; Gerlhone et al. 3.03% [23]; Toljanic et al. 3.92% [25]. Other mechanical complications were acrylic fracture (Francetti et al. 15% in the mandible, 19% in maxilla [21]; Ayna et al. 14.81% of acrylic suprastructures [24]) and metal framework fracture (*n* = 3) (Toljanic et al. 9.8% [25]; Francetti et al. 3% [21]). Queridinha et al. [28] reported the following complications: group 1 (axially and distally placed implants) (10% fracture of provisional prostheses, 3.33% attachment screw loosening, 6.66% abutment screw loosening, 3.33% abutment and an attachment screw loosening, 3.33% ceramic chipping); and group 2 (axial implants) (16.66% provisional prosthesis fracture, 3.33% abutment screw loosening, 3.33% attachment screw fracture, 3.33% ceramic chipping) [28].

Regarding biological complications, Hopp et al. [27] described the following implant complications: infection (0.67%); fistula (0.11%); mucositis (5.3%); peri-implant pathology (2.66%); and abscess (1.43%). Most of these complications were observed in tilted implants (182 versus 131 in axial implants) with a statistically significant difference [27].

Francetti et al. reported peri-implantitis affecting 1.53% of dental implants [21]. Queridinha et al. observed peri-implantitis in 8.3% of dental implants [28]. Notwithstanding, these authors did not differentiate between tilted and straight implants.

## 4. Discussion

The aim of this systematic review was to determine the long-term clinical behavior of axial implants in comparison with intentionally tilted dental implants supporting fixed restorations in both partially and totally edentulous arches. The review assessed implant behavior after at least 5 years of function, in terms of implant survival and success rates, peri-implant marginal bone loss (MBL), and prosthodontic/biological complications.

The studies included a total of 1113 patients, who received 3987 implants, of which 2036 were axial and 1951 were tilted. The results showed a high survival rate for both axial (mean SR 96.66%) and tilted implants (mean SR 96.93%). It should be considered that usually, authors do not include in their studies the population groups that present risk factors that could reduce survival rates such as diabetes, history of periodontal disease, poor hygiene, parafunctions, or limited bone volume [30]. Other factors that may alter intraoral health status, such as piercings, could also affect the implant survival rate [31].

Regarding implant success, mean rates of 95.1% and 96.36% were found for axial and tilted implants, respectively. It should be noted that the mean success rate (mean SR 96.36%) was virtually identical to the survival rate (mean SR 96.93%) for tilted implants. This might be explained by the fact that not all the reviewed studies reported success rates. In turn, as noted above, not all the studies applied the same criteria to evaluate implant success rates.

The present review obtained similar results to previous systematic reviews that have made long-term analyses of restorations supported by straight implants alone [32,33]. The use of tilted implants would appear to be a viable alternative to axial implants offering a number of advantages: they avoid extensive bone augmentation surgeries and make it possible to place long implants and so increase the bone-to-implant contact area and primary stability.

Regarding MBL, results of meta-analysis found MBL to be 0.113 mm higher around tilted implants in comparison with axial implants after >5 years of function, with no statistically significant difference (*p* = 0.369). According to Papaspyridakos et al. [34], it can be expected that marginal bone loss around implants will reach a maximum of 1.5 mm during the first year. The studies analyzed in the present review all reported marginal bone losses within this range (regarded as acceptable). These results agree with previous systematic reviews [13,35,36,37]. Monje et al. [13] concluded that marginal bone loss around tilted implants that were splinted to support fixed prostheses was not significantly different from straight implants in the short and medium term. Furthermore, Del Fabbro et al. [35] affirmed that this non-difference continues for up to 5 years of function but were unable to draw conclusions regarding the long term. Apaza Alccayhuaman et al. [36] found that MBL was 0.03 mm higher for tilted implants in a comparison to the behavior of tilted and straight dental implants in function for >3 years. As far as we are aware, the present systematic review is the first published paper to assess the longer-term behavior of tilted implants during a long follow-up of ≥5 years.

As for mechanical complications, most of the studies reviewed did not provide data on mechanical complication affecting axial or tilted implants separately since most prostheses were supported by both types of implants. Nevertheless, fracture of the resin base or teeth was one of the most commonly reported complications, particularly in full arch restorations. Framework fracture and screw loosening were also recorded by some authors. Francetti et al. [21] reported framework fracture in 3% of cases; Toljanic et al. [25] reported five framework fractures out of a total of 51 patients (9.8%).

Screw loosening was observed in a larger number of studies affecting 0.92–3.92% of implants [23,24,25,26]. In partial restorations, Barnea et al. [26] recorded two screw loosenings and Queridinha et al. [28] concluded that patients with prostheses supported by one tilted and one axial implant suffered more attachment screw loosening or abutment screw loosening than partial prostheses supported by two axial implants. Nevertheless, the review was unable to draw definitive conclusions about whether tilted implants suffer a higher percentage of mechanical complications than prostheses supported only by axial implants in full arch restorations. Insufficient evidence was found to confirm or reject the possibility that prostheses supported by tilted implants are associated with a greater number of mechanical complications. We agree with Apaza Alccayhuaman et al. [36] who expressed the need to carry out randomized controlled trials including only restorations supported by either straight or tilted implants, as this would allow a real comparison of the technical between them

In addition, it would also be useful to use advances in dentistry to accurately measure implant angulation and standardize the degree of angulation of implants to facilitate data comparison. Many novel scientific approaches are currently used in dentistry [38]. In implant surgery, a totally guided system using fixation screws with a flapless protocol has shown the greatest accuracy of all computer-aided implant surgery systems [39].

It should be noted that the present systematic review suffered some limitations:

- Only non-randomized clinical trials were included, while the comparative studies presented a high risk of bias [40].
- Concerning the restoration materials used in the different studies, the types of restoration placed were diverse and the complications were also diverse and irregularly distributed. Future research should also take the antagonist arch into consideration, as this factor in combination with the occlusion may influence the type and number of mechanical complications.
- Eight of the studies reported the degree of angulation of their tilted implants (20°, 30°, 40°, 45°, and 50°), but only three studies reported the measurement methods used to ensure these angulations (guide, splint, or intraoral x-ray). It would be useful to establish an effective method of accurately confirming that the implant angulation achieved is as intended. Future studies should compare the influence that the degree of implant angulation has on implant survival or success rates, MBL, and biomechanical complications.

Because of the limitations outlined above, the results of this review should be interpreted with caution. Nevertheless, according to the results of both qualitative and quantitative analyses, tilting the distal implant would appear to be a viable means of avoiding extensive regenerative surgeries when residual bone is scarce.

## 5. Conclusions

Within the limitations of this systematic review, it may be concluded that ITDI would appear to be a reliable option for supporting fixed restorations. Axial and tilted dental implants showed similar results in implant survival or overall implant success rates in the long term (>5 years), both in full arch and partial restorations. Moreover, no statistically significant differences were found in MBL loss. Therefore, using tilted dental implants when posterior bone is lacking and extensive bone augmentation surgeries are rejected by the patient may offer a valid therapeutic option for implant-supported restorations and should accordingly be known and jointly planned by dental practitioners, oral surgeons, and prosthodontists. However, some of the parameters that could possibly influence the results presented considerable heterogeneity: abutment procedures, dental implant length and width, loading times, and prosthetic complications. For this reason, further studies are needed with adequate protocols, sufficient sample sizes, and long-term follow-up periods in order to confirm the present results.

## Figures and Tables

**Table 1:** Information about studies reviewed, evaluating straight implants compared with tilted implants supporting fixed restorations: study, sample size, implant number, follow-up, location, and rehabilitation type.

Authors and Year	Study	Patients Number	Implants (*n*)	Implants Axial (*n*)	Implants Tilted (*n*)	Implants (Length and Width) (mm)	Follow-Up	Full Arch/Partial	Location (Maxilla/Mandible)
Francetti et al. 2012 [21]	Prospective Cohort Study	12 (47 total study)	48 (196 total study)	*n* = 24 (98 total study)	*n*= 24 (98 total study)	NR and 4	Maxilla: 22–40 months (mean 33.8 months). Mandible: 30–60 months (mean 52.8 months)	Full Arch-All on 4	Both
Agnini et al. 2014 [22]	Prospective Cohort Study	30 (total study)	52 (202 total study)	50 (165 total study)	2 (37 total study)	NR and ≥3, 7	18–67 months (mean 44 months)	Full Arch	Both
Gherlone et al. 2018 [23]	Prospective Cohort Study	29 (total study)	128 at 5 years (total study)	64 at 5 years (total study)	64 at 5 years (total study)	13 mm (axial); 13 and 15 mm (tilted). Axial (3.8, 4.5 mm); Tilted (4.5 mm)	5 year	Full Arch-All on 4	Both
Ayna et al. 2015 [24]	Prospective Cohort Study	27 (total study)	108	54	54	13 (axial); 15 (tilted) and 4	5 year	Full Arch-All on 4	Mandible
Toljanic et al. 2018 [25]	Retrospective Cohort Study	51 (total study)	86 at 5 years (total study 102)	35 (total study 38)	51 (total study 64)	NR and NR	Intervals 1, 3, 5 year	Full Arch-All on 4	Both
Barnea et al. 2016 [26]	Retrospective Cohort Study	13 (29 total study)	26 (58 total study)	13 (29 total study)	13 (29 total study)	11.5–16 and 3.75, 4.2	1–17 year (mean 4.86 year)	Partial	Posterior Maxilla
Hopp et al. 2017 [27]	Retrospective Cohort Study	891 (total study)	3419 at 5 years (total study 3564)	1706 at 5 years (total study 1782)	1713 at 5 years (total study 1782)	7–18 and 3.3, 3.75, 4, 5	5 year	Full Arch-All on 4	Maxilla
Queridinha et al. 2016 [28]	Retrospective Cohort Study	60 (total study)	120	90 at 5 yearss (total study)	30 at 5 years (total study)	7–18 and 3.75, 4.2	5 year	Partial	Posterior Maxilla

**Table 2:** Information about studies reviewed, evaluating straight implants compared with tilted implants supporting fixed restorations: study, number of restorations, loading, angulation of implants, abutment, marginal bone loss. (MBL: mean marginal bone loss; NR: Non Registered).

Authors and Year	Restorations (*n*)	Loading	Angulation of Implant	Abutment Y/N	MBL and Desviation MBL (Axial) (mm)	MBL and Desviation MBL (Tilted) (mm)
Francetti et al. 2012 [21]	12 (49 total study)	Inmediate	30°	Y (0°, 30°)	0.51 ± 0.17	0.39 ± 0.18
Agnini et al. 2014 [22]	8 (36 total study)	Inmediate	20–40°	Y (angulation not specified)	1.73 ± 0.14 (maxilla); 1.70 ± 0.18 (mandible)	2 (maxilla) ± 0.14
Gherlone et al. 2018 [23]	12 max; 20 mandible	Inmediate	25–30°	Y (30°)	1.08 ± 0.45 (maxilla); 1.04 ± 0.61 (mandible)	1.02 ± 0.67 (maxilla); 1.09 ± 0.56 (mandible)
Ayna et al. 2015 [24]	27	Inmediate	45°	Y (30°)	0.78 ± 0.10 (region 32); 0.78 ± 0.10 (region 42	1.24 ± 0.13 (region 35); 1.30 ± 0.13 (region 45)
Toljanic et al. 2018 [25]	51	Inmediate	NR	Y (angulation not specified)	0.14 ± 0.34	0.79 ± 1.42
Barnea et al. 2016 [26]	13 (29 total study)	Delayed	20–50°	Y (15–25°)	1.50 ± 0.81	1.50 ± 0.67
Hopp et al. 2017 [27]	891	Inmediate	30–45°	Y (0°, 17°, 30°)	1.14 ± 0.71	1.19 ± 0.82
Queridinha et al. 2016 [28]	60	Inmediate	30–45°	Y (30°)	2.11 ± 0.44	1.92 ± 0.48

**Table 3:** Information about studies reviewed, evaluating straight implants compared with tilted implants supporting fixed restorations: study, implant survival rate, success rate, and complications.

Authors and Year	Success of Axial Implants	Success of Tilted Implants	Survival of Tilted Implants	Survival of Axial Implants	Complications
Francetti et al. 2012 [21]	NR	NR	24/24 (100%)	24/24 (100%)	Three axial mandibular implants in two patients showed peri-implantitis; fracture of the acrylic prosthesis (15%) in the mandible and (19%) in the maxilla (19%); fracture of the framework after 3 years of loading (3%).
Agnini et al. 2014 [22]	NR	NR	37/37 (100%)	161/165 (97.57%)	Breaking of esthetic veneering of the temporary prostheses (5.5% of cases).
Gherlone et al. 2018 [23]	NR	NR	63//64 (98.44%)	64/64 (100%) (maxilla and mandible)	Prosthetic survival rate (100%). Occlusal screw loosening was observed in 3.03% of cases (4 implants) within 6 months of follow-up.
Ayna et al. 2015 [24]	100%	100%	54/54 (100%)	54/54 (100%)	Ceramic supra-structures: only a single loosening of a fixation screw/acrylic restorations: abrasion in all restorations neither esthetically nor functionally relevant, and 28.6 % veneer fractures.
Toljanic et al. 2018 [25]	NR	NR	44/51 (86.2%)	30/35 (85.71%)	Fracture resin base/teeth (*n* = 30); framework fracture (*n* = 5); abutment screw loose(*n* = 4); abutment fracture(*n* = 2); food impaction (*n* = 2); excessive occlusion(*n* = 2); seating error of angled abutment (*n* = 2); bulky construction (*n* = 1); fractured provisional (*n* = 1); speech problems (*n* = 1).
Barnea et al. 2016 [26]	26/29 (89.6%)	27/29 (93.1%)	NR	NR	Prosthetic survival rate was 100%. One bridge (3.4%) was de-cemented, and two screws loosening (3.4%) occurred in the same patient.
Hopp et al. 2017 [27]	76//1782 (95.7%)	69//1782 (96.1%)	NR	NR	Prosthesis survival rate (99.8%). Biological complications (313 implants in 209 patients (23.5%): infection (24), fistula (4), mucositis (189), peri-implant pathology (95), or abscess (*n* = 1). Biological complications were observed with 131 axial and 182 tilted implants.
Queridinha et al. 2016 [28]	NR	NR	NR	NR	Five patients presented biologic complications (8.3%) and sixteen patients presented mechanical complications.

**Table 4:** Quality assessment of studies reviewed using the Newcastle–Ottawa scale (NOS).

	Selection				Comparability		Exposure			Number of Stars (out of 9)
Study	S1	S2	S3	S4	C1	C2	E1	E2	E3	
Francetti et al. [21]	★	★	0	0	★	0	★	0	★	5
Agnini et al. [22]	★	★	★	0	★	0	★	0	0	3
Gherlone et al. [23]	★	★	★	0	★	0	★	0	★	6
Ayna et al. [24]	★	★	0	★	0	0	★	0	★	5
Toljanic et al. [25]	★	★	★	0	★	0	★	0	0	5
Barnea et al. [26]	★	★	★	0	★	0	0	★	0	5
Hopp et al. [27]	★	★	★	0	0	0	★	★	0	5
Queridinha et al. [28]	★	★	0	★	0	★	★	0	0	5
